# Adoption of World Health Organization Best Practices in Clinical Trial Transparency Among European Medical Research Funder Policies

**DOI:** 10.1001/jamanetworkopen.2022.22378

**Published:** 2022-08-01

**Authors:** Till Bruckner, Florence Rodgers, Lea Styrmisdóttir, Sarai Keestra

**Affiliations:** 1 Berlin Institute of Health at Charité–Universitätsmedizin Berlin, QUEST Center, Berlin, Germany; 2 TranspariMED, Bristol, United Kingdom; 3 Royal Cornwall Hospitals National Health Service Trust, Truro, United Kingdom; 4 Cochrane Sweden, Lund, Sweden; 5 Department for Epidemiology and Data Science, Amsterdam University Medical Centers, University of Amsterdam, the Netherlands

## Abstract

**Importance:**

Research funders can reduce research waste and publication bias by requiring their grantees to register and report clinical trials.

**Objective:**

To determine the extent to which 21 major European research funders’ efforts to reduce research waste and publication bias in clinical trials meet World Health Organization (WHO) best practice benchmarks and to investigate areas for improvement.

**Design, Setting, and Participants:**

This cross-sectional study was based on 2 to 3 independent assessments of each funder’s publicly available documentation and validation of results with funders during 2021. Included funders were the 21 largest nonmultilateral public and philanthropic medical research funders in Europe, with a combined budget of more than US $22 billion.

**Exposures:**

Scoring of funders using an 11-item assessment tool based on WHO best practice benchmarks, grouped into 4 broad categories: trial registries, academic publication, monitoring, and sanctions. Funder references to reporting standards were captured.

**Main Outcomes and Measures:**

The primary outcome was funder adoption or nonadoption of 11 policy and monitoring measures to reduce research waste and publication bias as set out by WHO best practices. The secondary outcomes were whether and how funder policies referred to reporting standards. Outcomes were preregistered after a pilot phase that used the same outcome measures.

**Results:**

Among 21 of the largest nonmultilateral public and philanthropic funders in Europe, some best practices were more widely adopted than others, with 14 funders (66.7%) mandating prospective trial registration and 6 funders (28.6%) requiring that trial results be made public on trial registries within 12 months of trial completion. Less than half of funders actively monitored whether trials were registered (9 funders [42.9%]) or whether results were made public (8 funders [38.1%]). Funders implemented a mean of 4 of 11 best practices in clinical trial transparency (36.4%) set out by WHO. The extent to which funders adopted WHO best practice items varied widely, ranging from 0 practices for the French Centre National de la Recherche Scientifique and the ministries of health of Germany and Italy to 10 practices (90.9%) for the UK National Institute of Health Research. Overall, 9 funders referred to reporting standards in their policies.

**Conclusions and Relevance:**

This study found that many European medical research funder policy and monitoring measures fell short of WHO best practices. These findings suggest that funders worldwide may need to identify and address gaps in policies and processes.

## Key Points

**Question:**

Do European medical research funders require grantees to register and report clinical trials in line with World Health Organization (WHO) best practices?

**Findings:**

This cross-sectional study of the 21 largest nonmultilateral public and philanthropic funders in Europe found that funders implemented a mean of 36% of WHO best practices in clinical trial transparency. Performance varied widely among funders, and some practices were more widely adopted than others.

**Meaning:**

These findings suggest that medical research funders in Europe could do more to accelerate medical progress and reduce research waste and publication bias.

## Teaser

This cross-sectional study examines rates of adoption of World Health Organization best practices in clinical trial transparency to reduce waste and publication bias.

## Introduction

According to the World Health Organization (WHO), a significant proportion of all clinical trials never make their results public.^1^ Not publishing clinical trial results in a timely manner constitutes research waste because nonpublic research results do not contribute to advancing science or improving clinical practice, and doing so risks the unnecessary duplication of research.^2^ Nonpublication of research results is associated with an estimated $85 billion of medical research funding being wasted annually across the world.^3^ In addition to leaving gaps in the scientific record,^4^ unregistered and unreported clinical trials may also be associated with a systematic distortion of the medical evidence base because positive trial outcomes are more likely to be made public than negative ones.^5^ This publication bias in the medical literature is associated with systematic overstatement of the efficacy and understatement of harms associated with drugs, medical devices, and nondrug interventions.^6^

The combination of evidence gaps and evidence distortion undermines regulatory decision-making, health technology assessment, clinical guideline development, public procurement, and decision-making on public health measures and individual treatment.^7^ For these reasons, prospective registration and publication of outcomes of all clinical trials constitute a global ethics requirement set out by the World Medical Association Declaration of Helsinki.^8^

Furthermore, for certain types of trials, prospective registration and timely uploading of outcome data onto trial registries are now legal requirements in several jurisdictions, including the European Union and United States.^9^ In addition to reduced research waste and publication bias, registry reporting requirements may be associated with accelerated medical progress. Outcomes typically have to be made public on registries within 12 months of a trial’s primary completion date, which is considerably faster than typical publication timelines in peer-reviewed medical journals.^10^

In 2017, WHO convened major medical research funders to sign up to the WHO joint statement on public disclosure of results from clinical trials (hereafter, *WHO Joint Statement*), which sets out global best practices in clinical trial registration and reporting.^11^ Signatories committed to reducing research waste in their portfolios by requiring their grantees to preregister trials on a WHO-linked trial registry and make their results public on the same registry within 12 months of trial completion. Funders also committed to monitoring grantees’ compliance with such policies, making these monitoring reports public, and imposing sanctions for noncompliance. A May 2022 World Health Assembly resolution called on funders to mandate trial registration and promote reporting of all trial results in line with WHO Joint Statement requirements.^12^

Experience suggests that medical researchers frequently fail to follow best practices in clinical trial registration and reporting, typically owing to a lack of awareness and oversight.^13,14^ Some funders have demonstrated that monitoring can add substantial value without antagonizing grantees or burdening them with excessive red tape.^14,15,16,17,18^ While implementing a monitoring system can be challenging for funders if their existing systems do not systematically capture data on all trials in their portfolios, these challenges can be overcome.^15^We are not aware of any barriers to adopting strong policies.

### Rationale

Previous research has found significant gaps between WHO Joint Statement benchmarks and policies adopted by major global medical research funders.^19,20^ However, the assessment framework used by these studies was loosely based on WHO Joint Statement policy items, and some of the findings may now be outdated.

### Objectives

The primary objective of this study was to assess the extent to which policies and monitoring systems of the largest public and philanthropic medical research funders (excluding multilateral funders) in Europe met global best practice benchmarks as set out by the WHO Joint Statement. The secondary objectives were to assess whether and how funder policies referred to the Consolidated Standards of Reporting Trials (CONSORT) reporting guideline for clinical trial reporting in journals.^21^ While more than 500 journals have endorsed the CONSORT guideline,^22^ it is not mentioned explicitly in the WHO Joint Statement.

## Methods

QUEST determined that ethics approval was not required for this cross-sectional study given that our assessments were based on publicly available institutional data. QUEST waived a requirement for informed consent because all respondents were contacted in their capacity as officially designated institutional spokespersons. Study design and reporting were performed in accordance with the Strengthening the Reporting of Observational Studies in Epidemiology (STROBE) reporting guideline for cross-sectional studies.

A peer-reviewed database of the world’s largest noncommercial medical research funders was used to select the study cohort.^23,24^ Funders with an annual expenditure on health research of $50 million or more (all budget figures in 2013 US dollars) were included regardless of whether they funded extramural or intramural research or both. Funders were excluded if they were partially or wholly geographically located outside of the European continent or were multinational funders (ie, the European Union and its various programs, such as Horizon-2020). We also excluded Ludwig Institute for Cancer Research, a US-based institution^25^ mistakenly identified as UK based in the list; the Innovative Medicines Initiative, which is a public-private partnership^26^; and the UK Engineering and Physical Sciences Research Council and the UK Foreign, Commonwealth and Development Office, which do not directly fund clinical trials (although the latter does so through intermediaries). The German Federal Ministry of Health was retained because it funded at least 1 COVID-19 trial.^27^ The final cohort comprised the 21 largest nonmultilateral public and philanthropic funders in Europe, which have a combined annual medical research budget of more than $22 billion. Of these, 7 funders have signed up to the WHO Joint Statement: Institut National de la Santé et de la Recherche Médicale (INSERM), Institut Pasteur, the UK Medical Research Council, the UK National Institute for Health Research, Research Council of Norway, Wellcome Trust, and the Netherlands Organisation for Health Research and Development (ZonMw).

Scoring of funders was done using an 11-item assessment tool based on WHO Joint Statement benchmarks, with a range of possible scores from 0 to 11 points. The 11 items were grouped into 4 broad categories: trial registries, academic publication, monitoring, and sanctions (Table). An additional item captured whether and how funders referred to CONSORT, but this item was not scored. Before rollout, the tool was piloted on 3 funders (the Research Council of Norway, Swedish Research Council, and Wellcome Trust). Supplementary online searches done via search engine (Google) and using a list of predetermined key words in English and the national language of the funder were also piloted but were subsequently discontinued given that this search did not detect additional policies (this was a deviation from protocol). After the pilot phase, we registered the study with the Open Science Framework (Y6NDH). The protocol, assessment tool, rater guide, search terms (discontinued), adjudication tracker, individual and consolidated score sheets, aggregated data sets, archived funder policies, and correspondence with funders are available on Github.^28^

**Table.** WHO Best Practices in Clinical Trial Transparency

Domain	Scoring item	WHO verbatim wording
Registries	Prospective trial registration	“Before any clinical trial is initiated (at any Phase) its details must be registered in a publicly available, free to access, searchable clinical trial registry complying with WHO’s international agreed standards The clinical trial registry entry must be made before the first subject receives the first medical intervention in the trial (or as soon as possible afterwards).”
	Registry records kept up to date	“Clinical trial registry records should be updated as necessary to include final enrolment numbers achieved and the date of primary study completion (defined as the last data collection time point for the last subject for the primary outcome measure). If clinical trials are terminated, their status should be updated to note the date of termination and to report the numbers enrolled up to the date of termination.”
	Results posted to registry within 12 mo	“We will work towards a time frame of 12 months from primary study completion (the last visit of the last subject for collection of data on the primary outcome) as the global norm for summary results disclosure.”
	Protocol posted to registry within 12 mo	“Access to a sufficiently detailed clinical trial protocol is necessary in order to be able to interpret summary results. Therefore, we also encourage development of requirements that the protocols are made publicly available no later than the time of the summary results disclosure as part of the clinical trial registry summary results information (including amendments approved by ethics committees/institutional review boards and either as uploaded electronic document formats, such as PDFs or links to the PDF).”
Journals	Results made public in journal	“Publication in a journal is also an expectation, with an indicative time frame of 24 months from study completion to allow for peer review, etc.”
	Trial identification included in all publications	“The Trial ID or registry identifier code/number should be included in all publications of clinical trials and should be provided as part of the abstract to PubMed and other bibliographic search databases for easy linking of trial-related publications with clinical trial registry site records.”
	Open access publication	“We are all supporters of open access policies and consider that publications describing clinical trial results should be open access from the date of publication, wherever possible.”
Monitoring	Funder monitors trial registration	“We each agree to monitor registration and endorse the development of systems to monitor results reporting on an ongoing basis.”
	Funder monitors results reporting	
Sanctions	Funder considers PI past reporting record	“Reporting of previous trials realises the value of funding; therefore, the contribution made from reporting previous trials, whatever their results, will be considered in the assessment of a funding proposal. When a PI applies for new funding, they may be asked to provide a list of all previous trials on which they were PI within a specified time frame and their reporting status, with an explanation where trials have remained unreported.”

Abbreviations: ID, identification; PI, principal investigator; WHO, World Health Organization.

At least 2 researchers among F.R., L.S., and S.K. searched each funder website between February and May 2021 and filled out score sheets independently, with a rater guide supporting consistency across reviewers. Scoring was binary (yes or no); policies that did not cover all interventional trials or were nonbinding received no scores but were captured in separate columns in assessment sheets. In all cases, the hyperlink to the policy and relevant policy text were extracted and archived on Github. A separate team member (T.B.) reviewed, verified, and consolidated ratings. Borderline cases were resolved through team discussion and documented. Inter-rater reliability was not assessed given that the aim was to capture all relevant policy statements.

### Statistical Analysis

The press departments of all 21 funders were contacted 3 times by email with a copy of the score sheet and supplemental materials and invited to mark possible errors and omissions. Responses were received from 15 funders, and 14 funders provided specific feedback. Based on this feedback, ratings were adjusted for 18 of 154 items (ie, 14 funders × 11 items = 154 items). Among these 18 items, 9 ratings adjustments affected funder scores, of which 8 adjustments involved monitoring and sanctioning processes that were not publicly visible on funder websites. In 4 cases, the team decided not to make changes proposed by a funder; an overview of changes made and declined is provided in the eFigure in the Supplement. A third rater (F.R. and S.K.) independently reassessed 7 funders that had not substantively responded by November 2021; no additional policy items were detected.^29^

## Results

**Figure 1.**
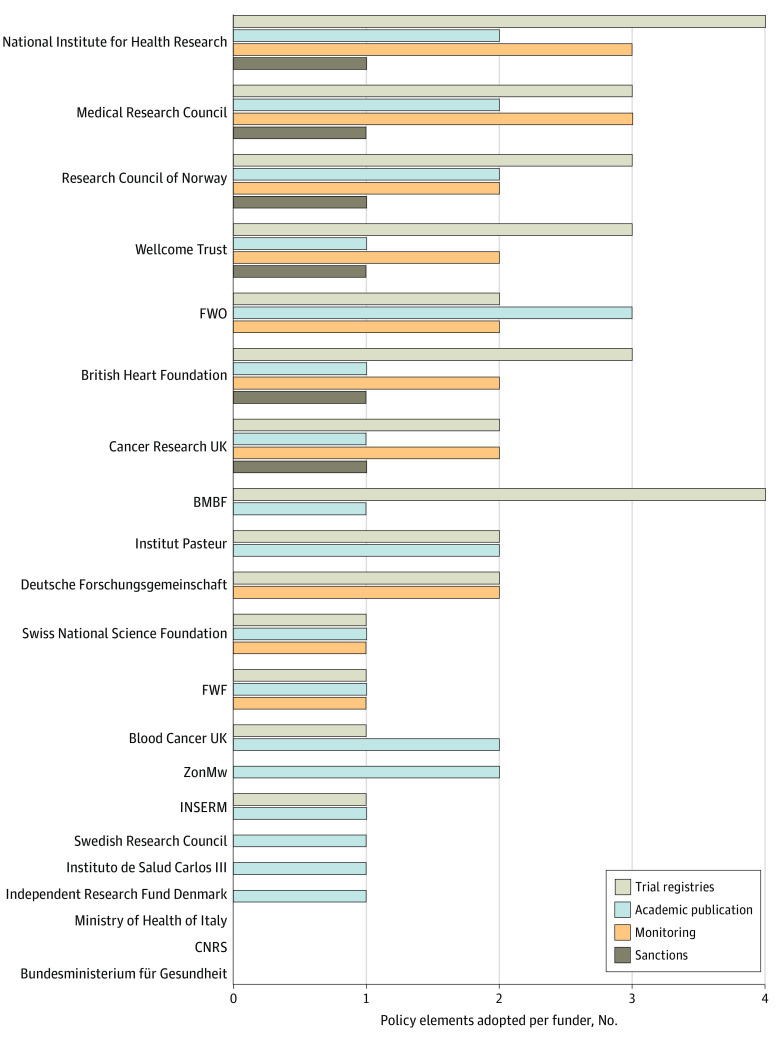
Policy Elements Adopted per Funder The maximum number of elements is 11. Items are grouped into 4 domains: trial registries, academic publication, monitoring, and sanctions. BMBF indicates the German Federal Ministry of Education and Research; CNRS, the French Centre National de la Recherche Scientifique; FWF, Austrian Science Fund; FWO, Research Foundation-Flanders; INSERM, Institut National de la Santé et de la Recherche Médicale; ZonMw, Netherlands Organisation for Health Research and Development.

Figure 1 displays the adoption of WHO best practices across 21 funders. Some best practices were more widely adopted than others. There were 16 funders (76.2%) that already explicitly required trial results to be published in open access journals. The WHO Joint Statement strongly focused on registration and reporting on trial registries. Although 14 funders (66.7%) mandated prospective trial registration, 6 funders (28.6%) required that trial results be made public on these registries within 12 months of trial completion. Less than half of funders actively monitored whether trials were registered (9 funders [42.9%]) or whether results were made public (8 funders [38.1%]). Less than one-third of funders (6 funders [28.6%]) made monitoring reports public. There were 3 funders (14.3%) who envisaged imposing sanctions for noncompliance with their policies.

**Figure 2.**
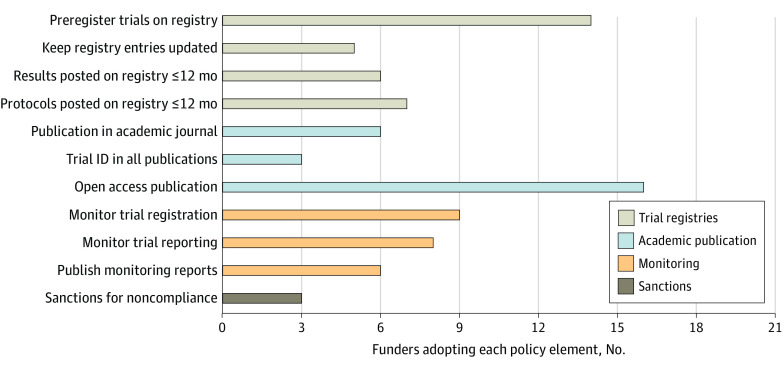
Funders Adopting Specific Policy Elements The maximum number of funders is 21. Items are grouped into 4 domains: trial registries, academic publication, monitoring, and sanctions ID indicates identification.

European funders implemented a mean of 4 of 11 best practices in clinical trial transparency set out by WHO (36.4%). The extent to which funders adopted WHO best practice items varied widely, ranging from 0 practices for the French Centre National de la Recherche Scientifique and the ministries of health of Germany and Italy to 10 practices (90.9%) for the UK National Institute of Health Research (Figure 2).

This scoring reflects only mandatory policy items that covered all clinical trials. In 18 instances, funders encouraged a practice but did not make it compulsory; notably, Deutsche Forschungsgemeinschaft encouraged 5 practices without mandating them. In 3 instances funder policy items applied to a limited subset of trials. An overview of nonbinding and noncomprehensive policies is provided in the eFigure in the Supplement.

In total, 9 funders referred to CONSORT reporting standards within their policies. We found that 3 funders unambiguously mandated that grantees must report in line with CONSORT, while 6 funders encouraged use of CONSORT or noted it as a useful resource (eTable 1 in the Supplement).^19^

## Discussion

To our knowledge, this cross-sectional study provides the first comprehensive assessment of medical research funder clinical trial policies that is exclusively based on WHO best practice benchmarks as outlined in the Joint Statement. Independent rating by at least 2 researchers, review and consolidation by a third researcher, transparent adjudication, and respondent validation by two-thirds of assessed funders strengthened data quality. Our reproducible methods and tools may be used to reassess the same cohort to document changes over time, assess new funder cohorts, and (with slight modifications) assess clinical trial policies of commercial and noncommercial trial sponsors rather than medical research funders. Archiving of all project tools and documentation on GitHub enables independent replication, including with other cohorts.

This study found that adoption of measures to reduce research waste and reporting bias in clinical trials by most public and philanthropic medical research funders in Europe fell significantly short of WHO best practices. The 21 largest funders implemented a mean of 4 of 11 best practices (36%) in clinical trial transparency set out by WHO. However, performance varied widely among individual funders, ranging from 0 to 10 best practices.

Notably, 7 funders still did not require their grantees to prospectively register clinical trials, although this is a long-standing global ethics requirement. Furthermore, 15 funders still did not require rapid outcome sharing via trial registries, thereby missing an opportunity to implement a requirement associated with improved data comprehensiveness,^30^ decreased research waste, and accelerated medical progress^31^ but not decreased opportunities to subsequently publish the same results in a peer-reviewed journal.^32^ These funders’ failures to adopt and enforce relevant policies are associated with the generation and perpetuation of well-documented fiduciary, public health, and personal health risks.

We are aware of 2 instances in which there may be drawbacks associated with adopting WHO best practices. First, the requirement to publish trial results in a peer-reviewed journal may be inappropriate in the case of trials terminated early with few participants; in such cases, funders may wish to exercise discretion and require results to be made public only in a trial registry so that they may be integrated into future meta-analyses. Second, considering researcher past trial registration and reporting track records when reviewing novel grant applications (as recommended by the WHO Joint Statement) is arguably a suboptimal sanctions mechanism; focusing on institutional rather than individual track records may be more appropriate, efficient, and effective.^9^

After our outreach to funders, Wellcome Trust strengthened its policies^33^ and INSERM^34^ and the Swedish Research Council^35^ adopted new polices, which may have been informed by our outreach; these funders would score significantly higher if reassessed today. Several other funders indicated that they would take into account our data during upcoming policy reviews (eAppendix in the Supplement). To aid these developments, we have created a template policy document with example texts for each policy item to facilitate the adoption of WHO best practices by funders (eTable 2 in the Supplement).

### Limitations

This study has several limitations. Our cohort selection relied on funding data compiled in 2013; to the best of our knowledge, there are no more recent data sets ranking funders by funding volume. We do not provide data on non-European, multilateral, or commercial research funders. Of 21 funders, 7 funders did not respond to our outreach. As a result, relevant items for those funders may have been missed, especially if relevant policy items were not publicly accessible online. We did not reassess funders that strengthened their policies after assessment. Our scoring criteria only partially overlap with those of a 2018 assessment that covered 10 funders in our cohort,^19^ so we were unable to document changes over time.

## Conclusions

Responses by medical research funders to outreach in this cross-sectional study suggest considerable willingness among some funders in Europe do more to accelerate medical progress and decrease research waste and publication bias. Every policy element we assessed has already been implemented by at least 3 funders, suggesting feasibility. Funders worldwide may be able to use our scoring tool to identify and address gaps in their policies and processes. We have created a template policy document with example texts for each policy item to facilitate the adoption of WHO best practices by funders (eTable 2 in the Supplement).

## Notes

Supplement. **eFigure.** Rating Changes and Partial Policies **eTable 1.** Funder Mentions of Consolidated Standards of Reporting Trials (CONSORT) **eTable 2.** Template Funder Policy Document **eAppendix.** Planned Reviews and Changes of Funder Policies
