# Supplementary material for: Adoption of World Health Organization Best Practices in Clinical Trial Transparency Among European Medical Research Funder Policies
Source: JAMA Netw Open. 2022 Aug 1;5(8):e2222378. doi: 10.1001/jamanetworkopen.2022.22378 (PMC9344358; doi:10.1001/jamanetworkopen.2022.22378)
Supplement: Supplement. — eFigure. Rating Changes and Partial Policies eTable 1. Funder Mentions of Consolidated Standards of Reporting Trials (CONSORT) eTable 2. Template Funder Policy Document eAppendix. Planned Reviews and Changes of Funder Policies [file jamanetwopen-e2222378-s001.pdf]

# Supplemental Online Content

Bruckner T, Rodgers F, Styrmisdóttir L, Keestra S. Adoption of World Health Organization best practices in clinical trial transparency among European medical research funder policies. *JAMA Netw Open*. 2022;5(8):e2222378. doi:10.1001/jamanetworkopen.2022.22378

**eFigure.** Rating Changes and Partial Policies

**eTable 1.** Funder Mentions of Consolidated Standards of Reporting Trials (CONSORT)

**eTable 2.** Template Funder Policy Document

**eAppendix.** Planned Reviews and Changes of Funder Policies

This supplemental material has been provided by the authors to give readers additional information about their work.

**eFigure.** Rating Changes and Partial Policies

The following table provides an overview of:

(a) of non-comprehensive and non-binding funding policies, which did not receive any points in the scoring system used by the study

(b) changes made and declined by the study team based on feedback received from sponsors

| Funder | Country | WHO | Pre-register | Update | R-Results | Protocol | Journal pub | Trial ID | OA pub | Monitor reg | Monitor rep | M-reports | Sanctions | DeVito |
|---|---|---|---|---|---|---|---|---|---|---|---|---|---|---|
| Blood Cancer UK | UK | | YES | NO | NO | NO | YES | NO | YES | NO | NO | NO | NO | |
| BMBF | Germany | | YES | YES | YES | YES | YES | NO | S | NO | NO | NO | NO | YES |
| British Heart Foundation | UK | | YES | S | YES | YES | NO | S | YES | YES | YES | YES | NO | |
| Bundesministerium für Gesundheit | Germany | | NO | NO | NO | NO | NO | NO | NO | NO | NO | NO | NO | |
| Cancer Research UK | UK | | YES | NO | YES | NO | NO | S | YES | YES | YES | YES | S | |
| CNRS | France | | NO | NO | NO | NO | NO | NO | NO | NO | NO | NO | NO | YES |
| Independent Research Fund Denmark | Denmark | | NO | NO | NO | NO | NO | NO | YES | NO | NO | NO | NO | |
| Deutsche Forschungsgemeinschaft | Germany | | YES | S | S | YES | S | NO | S | YES | YES | NO | S | YES |
| FWO | Belgium | | YES | NO | YES | NO | YES | YES | YES | YES | NO | NO | YES | |
| FWF | Austria | | YES | NO | NO | NO | NO | NO | YES | YES | NO | NO | NO | |
| INSERM | France | YES | YES | NO | NO | NO | NO | NO | YES | NO | NO | NO | NO | YES |
| Institut Pasteur | France | YES | YES | YES | NO | NO | YES | NO | YES | NO | NO | NO | NO | |
| Instituto de Salud Carlos III | Spain | | NO | NO | NO | NO | NO | NO | YES | NO | NO | NO | NO | YES |
| Medical Research Council | UK | YES | YES | YES | P | YES | NO | YES | YES | YES | YES | YES | YES | YES |
| Ministry of Health of Italy | Italy | | NO | NO | NO | NO | NO | NO | NO | NO | NO | NO | NO | YES |
| National Institute for Health Research | UK | YES | YES | YES | YES | YES | YES | S | YES | YES | YES | YES | YES | YES |
| Research Council of Norway | Norway | YES | YES | YES | S | YES | S | YES | YES | YES | YES | YES | NO | |
| Swedish Research Council | Sweden | | NO | NO | NO | NO | NO | NO | YES | NO | NO | NO | NO | |
| Swiss National Science Foundation | Switzerland | | YES | NO | NO | S | NO | NO | YES | NO | YES | NO | S | |
| Wellcome Trust | UK | YES | YES | P | YES | YES | S | NO | YES | YES | YES | YES | P | YES |
| ZonMw | Netherlands | YES | S | NO | NO | S | YES | NO | YES | NO | NO | NO | NO | YES |

S = funder supports/encourages practice without mandating it
P = funder policy item only applies to a specific sub-set of trials (but not to all trials)
BLUE HIGHLIGHTING = change made in response to funder feedback
BLACK HIGHLIGHTING = funder requested change but original score maintained

*Notes:*
*The column "WHO" identifies funders that have signed up to the WHO Joint Statement.*
*The column "DeVito" notes where a funder had previously been assessed by DeVito et al 2018 using a different methodology.*

© 2022 Bruckner T et al. *JAMA Network Open.*

**eTable 1.** Funder Mentions of Consolidated Standards of Reporting Trials (CONSORT)

| | Funder | Text referring to CONSORT |
|---|---|---|
| Mandatory | DFG (Germany) | *Grant application guidance for trials:* "The DFG expects compliance with existing reporting guidelines (e.g. www.equator-network.org). Please indicate which of these guidelines will be followed." |
| | INSERM (France) | From 2022 onwards, PIs will also have to respect the Consort Statement, as well as an external audit in addition to the annual review of obligations. |
| | MRC (UK) | The MRC requires researchers that we fund to follow relevant reporting guidelines that have been developed to improve the design, analysis and reporting of experiments, such that readers can assess the robustness of the findings and reproduce the work if they wish. Examples are the ARRIVE guidance for animal research and CONSORT for clinical trials. |
| Recommended or flagged | BHF (UK) | Research results should be reported in accordance with the recommendations in the CONSORT statement or an alternative reporting guideline. |
| | CRUK (UK) | CONSORT listed under *"25 resources to help you develop a great population research proposal"* |
| | FWO (Belgium) | For clinical trials, please follow the CONSORT guidelines when reporting your study |
| | NIHR (UK) | *NIHR documents repeatedly use the following language:* "we advise you refer to the CONSORT statement and website for guidance" |
| | Wellcome (UK) | *This passage refers only to publications in the in-house journal Wellcome Open Research:* "For reporting of the intervention methodology itself, Wellcome Open Research endorses the TIDieR checklist, an extension of the CONSORT statement. (…) Articles in Wellcome Open Research must comply with consensus-based minimum reporting guidelines for research. Comprehensive lists of available reporting guidelines can be found on the EQUATOR network website for health research. Checklists are available for a number of reporting guidelines, including: Randomized controlled trials (CONSORT)..." |
| | ZonMW (NL) | When reporting about your studies, it is strongly recommended, and required for animal studies, to adhere to reporting guidelines for health research studies of the EQUATOR network. (...) The reporting guidelines for health research of the EQUATOR network provide researchers with a structured list of information to help them to ensure that the results of a scientific manuscript can be replicated and the manuscript is suitable for inclusion in systematic reviews. |

© 2022 Bruckner T et al. *JAMA Network Open.*

**eTable2.** Template Funder Policy Document

| Domain | Scoring item | WHO Joint Statement wording[1] | Sample funder policy texts |
|---|---|---|---|
| **REGISTRIES** | Prospective trial registration | "Before any clinical trial is initiated (at any Phase) its details must be registered in a publicly available, free to access, searchable clinical trial registry complying with WHO's international agreed standards (www.who.int/ictrp). The clinical trial registry entry must be made before the first subject receives the first medical intervention in the trial (or as soon as possible afterwards)." | "Registration of clinical trial in a public registry that complies with the WHO requirements before the inclusion of the first participant in the trial" (Institut Pasteur)<br><br>"The Grantholder must register any CRUK-funded or endorsed trial on a recognised trials registry such as the ISRCTN registry or the ClinicalTrials.gov register before the first patient is recruited. The Grantholder must notify CRUK of the registration number no later than the time of the subsequent scientific milestone report." (CRUK) |
| | Registry records kept up to date | "Clinical trial registry records should be updated as necessary to include final enrolment numbers achieved, and the date of primary study completion (defined as the last data collection timepoint for the last subject for the primary outcome measure). If clinical trials are terminated, their status should be updated to note the date of termination, and to report the numbers enrolled up to the date of termination." | "All relevant fields in the selected registry must be completed and updated on a regular basis (at least annually) until the study has been completed and the results published or made publicly available. Approved changes to the study protocol must be updated in the registry as soon as possible. If the study is terminated before the scheduled end-date, the registry must be updated with information on the date of termination and the number of participants in the study at the time of termination." (RCN)<br><br>"Registries must be updated during the study and key outcomes and trial protocols are to be made publicly available within 12 months from Primary Study Completion. Registry information should be updated regularly as appropriate and in line with instructions from the relevant NIHR secretariat/monitoring team and ISRCTN (or relevant registry) guidelines, including any changes to recruitment data and key outcomes." (NIHR) |
| | Results posted onto registry within 12 months | "We will work towards a timeframe of 12 months from primary study completion (the last visit of | "Results for all studies, irrespective of their outcomes, must be made available on the |

[1] Sources: https://www.who.int/news/item/18-05-2017-joint-statement-on-registration & https://www.who.int/docs/default-source/clinical-trials/ictrp-jointstatement-2017.pdf?sfvrsn=adad9dc4_2

| Domain | Scoring item | WHO Joint Statement wording[1] | Sample funder policy texts |
|---|---|---|---|
| | | the last subject for collection of data on the primary outcome) as the global norm for summary results disclosure." | trial registry within a year of the end of data collection."[2] (BMBF)<br><br>"Summarized results should be completed within 12 months of completion." *[Followed by an excellent retroactive passage: "Beneficiaries of FWO grants must also deposit all clinical trials that have not yet been registered and / or missing data and results in a public clinical register."]* (FWO) |
| | Protocol posted onto registry within 12 months | "Access to a sufficiently detailed clinical trial protocol is necessary in order to be able to interpret summary results. Therefore we also encourage development of requirements that the protocols are made publicly available no later than the time of the summary results disclosure as part of the clinical trial registry summary results information (including amendments approved by ethics committees/institutional review boards, and either as uploaded electronic document formats such as pdfs or links to the pdf)." | "The MRC requires all funded studies to make the study protocol, analysis plan and all relevant statistical analyses publicly available prior to the start of the study. For clinical trials, the protocol (or information about where to find the protocol) must be added to the registry entry within 12 months of the trial start." (MRC)<br><br>"The RCN expects the study protocol and Statistical analysis plan (SAP) to be made publicly available prior to the start of the study. Furthermore, information on how this information can be obtained must be included in the registry. When registering ClinicalTrials.gov, this information can be entered in the summary field." (RCN) |
| **JOURNALS** | Results made public in journal | "Publication in a journal is also an expectation, with an indicative timeframe of 24 months from study completion to allow for peer review etc." | "The grant recipient must publish with respect to the results of the project, unless publication would not serve any public interest whatsoever. The grant recipient must also publish with respect to projects that are terminated prematurely or that do not lead to the desired result. Publishing is a mandatory component of the project's activities." (ZonMW)<br><br>"Transparency regarding clinical trials therefore includes… the publication of a clinical research report in a (specialized) journal describing the design, the methods used and the results." (FWO) |

[2] Original in German: "*Zudem müssen zu alle Studien, unabhängig von ihrem Ergebnis, innerhalb von einem Jahr nach Schließen der Datenbank die Ergebnisse im Register eingestellt werden.*"

© 2022 Bruckner T et al. *JAMA Network Open.*

| Domain | Scoring item | WHO Joint Statement wording[1] | Sample funder policy texts |
|---|---|---|---|
| | Trial ID included in all publications | "The Trial ID or registry identifier code/number should be included in all publications of clinical trials, and should be provided as part of the abstract to PubMed and other bibliographic search databases for easy linking of trial related publications with clinical trial registry site records." | "The registration number for the study must be included in all publications from the study and must be specified in the article summary/abstract." (RCN)<br><br>"The relevant clinical registry and identifier will also be included in all specialist publications about the clinical trial in question in order to simplify the link between professional publications (detailing research method and results) and the public registry." (FWO) |
| | Open access publication | "We are all supporters of open access policies, and consider that publications describing clinical trial results should be open access from the date of publication, wherever possible." | "A condition for receiving a grant from us is that you publish your research articles so that they are available to all, free of charge on the internet. We only accept articles that are published with open access in your reporting on grants." (SCR)<br><br>"Published scientific articles, which are the result of full or partial financing by funds and foundations, must be made freely available to everybody via Open Access with the permission of the magazine." (DFF) |
| **MONITORING** | Funder monitors trial registration | "We each agree to monitor registration and endorse the development of systems to monitor results reporting on an ongoing basis." | N/A Useful guidance here |
| | Funder monitors results reporting | "We each agree to monitor registration and endorse the development of systems to monitor results reporting on an ongoing basis." | N/A Useful guidance here |
| | Monitoring reports made public | "We agree that transparency is important and therefore the outputs from the monitoring process will be publicly available." | Examples of monitoring reports linked below:<br>MRC, Wellcome, NIHR, CRUK |
| **SANCTIONS** | Funder considers PIs' past reporting record | "Reporting of previous trials realises the value of funding; therefore the contribution made from reporting previous trials, whatever their results, will be considered in the assessment of a funding proposal. When a PI applies for new funding, they may be asked to provide a list of all previous trials on which they were PI within a | "Past Registration and publication practice will be taken into account when reviewing applications for funding of new Clinical Trials… When submitting an application to NIHR programmes for funding for a new clinical trial, the applicant must disclose past publication and trial Registration history for any relevant publications and research grants held, referenced in the application. This policy is considered good practice and |

| Domain | Scoring item | WHO Joint Statement wording[1] | Sample funder policy texts |
|---|---|---|---|
| | | specified timeframe and their reporting status, with an explanation where trials have remained unreported.” | retrospective compliance is encouraged, although it will not be viewed as mandatory. NIHR will consider previous Registration and publication amongst a range of other criteria when making funding decisions.” (NIHR)<br><br>"New awards to applicants who have not reported findings from a previous MRC-funded trial will not commence until the earlier trial has been reported" (MRC)<br><br>“If you fail to comply with this policy, we will consider appropriate sanctions. These may include suspending your grant or not accepting new grant applications from you.” (Wellcome)<br><br>"The registration of clinical trials must be reported to the FWO during the project period and the completeness of the registration can be taken into account by the expert panels in deliberations about the allocation of future grant applications” (FWO1)<br>“Beneficiaries of FWO grants must also deposit clinical trials that were not yet registered and / or fill out missing data and trial outcome(s) in a public clinical register." (FWO2) |
| **OTHER*** | Require results sharing plan | “at the time of the initial grant submission, the plan for public disclosure of results should be included” | “When you register you must include a data sharing plan as part of the trial registration, in line with the 2017 International Committee of Medical Journal Editors (ICMJE) requirements on data sharing statements for clinical trials… When you apply for Wellcome funding for a clinical trial, you must submit an outputs management plan with your grant application. This should describe how and when trial data will be made more widely available” (Wellcome) |
| | Cover compliance costs | Compliance costs made “a cost eligible item in clinical trial budgets” | “We will meet the costs associated with registering the trial. You can ask for these as part of your grant application… Wellcome supports researchers to meet our data sharing requirements by funding |

© 2022 Bruckner T et al. *JAMA Network Open.*

| Domain | Scoring item | WHO Joint Statement wording[1] | Sample funder policy texts |
|---|---|---|---|
| | | | reasonable costs to prepare, store, and access clinical data in ways that are aligned to the FAIR principles. We encourage researchers to consider these costs in their outputs management plans at the application stage, but we may also be able to support unforeseen additional costs at a later stage if requested, for example the anonymisation of identifiable data, or repository fees." (Wellcome) |
| **CONSORT**** | n/a | n/a | "The MRC requires researchers that we fund to follow relevant reporting guidelines that have been developed to improve the design, analysis and reporting of experiments, such that readers can assess the robustness of the findings and reproduce the work if they wish. Examples are the ARRIVE guidance for animal research and CONSORT for clinical trials." (MRC)<br><br>"Proposals are assessed according to internationally accepted standards. For clinical trials, please follow the CONSORT guidelines when reporting your study (http://www.consort-statement.org/)." (FWO) |

** The WHO Joint Statement contains these two items, but they were not scored in this assessment.*
*** The WHO Joint Statement does not mention CONSORT.*

## **eAppendix.** Planned Reviews and Changes of Funder Policies

**Cancer Research UK**
New policy to be published in late 2021. Expected to include mandatory requirements to keep registry records up to date and upload protocols onto registry, which would increase CRUK's score to 8/11.

**FCDO**
FCDO is considering requiring grant-giving organisations that receive UK international development funding to publish trial registration and reporting monitoring reports. Early stage deliberations, no decision yet.

**Inserm**
Inserm adopted a new policy in July 2021 that substantially strengthens its policies to an approximate score of 9-11 out of 11 possible points (see scoresheet and correspondence with Inserm for details). Inserm will publish its first monitoring report in 2022.

**Medical Research Council**
MRC changed some online guidance to clarify that it requires prospective trial registration and the publication of results in journals. (These policies already existed but were not consistently communicated on the website at the time of assessment, see scoresheet and correspondence.)

**NIHR**
NIHR plan to review their policy in 2022 and may then extend the requirement to include the trial ID in publications from some trials to all clinical trials.

**Research Council of Norway**
RCN informed us that they already monitor results reporting and will make their first monitoring report public in 2022. Our scores already reflect these developments as NRC has concrete, time bound plans in this regard (see adjudication decision).

**Swedish Research Council**
The Swedish Research Council took into account our assessment during a 2021 policy review process. See correspondence. We did not re-asses the funder's new policies, which were published in January 2022, but they appear to be more strongly aligned with WHO best practices.

**Wellcome Trust**
Wellcome Trust in strengthened some policies in response to our outreach. According to correspondence, Wellcome since April 2021 mandates keeping registry data up to date and including the trial ID in publications. While we did not re-assess the new policies, these changes if confirmed would increase Wellcome's score to 9/11. (Wellcome also changed its "language around the sharing of results more explicit to encourage publication in a peer-reviewed journal, publishing platform or preprint server".)

**ZonMw**
ZonMW plans to review its (lack of a) requirement to make trial results public on trial registries after the launch of the new European CTIS registry at the end of January 2022.

© 2022 Bruckner T et al. *JAMA Network Open.*
